# A preclinical study comparing single- and double-root 3D-printed Ti–6Al–4V implants

**DOI:** 10.1038/s41598-023-27712-2

**Published:** 2023-01-17

**Authors:** Inna Chung, Jungwon Lee, Ling Li, Yang-Jo Seol, Yong-Moo Lee, Ki-Tae Koo

**Affiliations:** 1 Department of Periodontology and Dental Research Institute, School of Dentistry, Seoul National University, 101 Daehak-ro, Jongno-gu, Seoul, 03080 Korea; 2 Department of Periodontology, Seoul National University Dental Hospital, Seoul, Korea; 3 One-Stop Specialty Center, Seoul National University Dental Hospital, Seoul, Korea

**Keywords:** Biotechnology, Medical research

## Abstract

Recently, double-root implants have been investigated using 3D-printed technology. Here, we investigated damping capacity, microcomputed tomographic (micro-CT) and histological analyses of double-root 3D-printed implants compared with single-root 3D printed implants. Single- and double-root 3D-printed implants were fabricated and placed at both sides of mandibular third and fourth premolars in four beagle dogs. The damping capacity was measured, and periapical X-rays were taken every 2 weeks for 12 weeks. The bone volume/tissue volume (BV/TV) and bone mineral density (BMD) around the implants were measured with micro-CT. Bone-to-implant contact (BIC) and bone area fraction occupancy (BAFO) were measured in histological samples. The implant stability values between the groups were not significantly different, except at 4 and 12 weeks. The marginal bone changes were similar at the mesial and distal areas between the groups. The BV/TV and BMD values of the double-root 3D-printed implants showed no statistical difference through micro-CT analysis, but the double-root 3D-printed implants showed lower BIC and BAFO values through histomorphometric analysis compared to the single-root 3D-printed implants. Compared to single-root implants, 3D-printed double-root implants demonstrated comparable stability and bone remodeling around the fixtures, but the statistically significant bone loss in the furcation area remains problematic.

## Introduction

With the recent increase in the elderly population, those in need of dental rehabilitation in edentulous areas are also increasing^1^. Research on implant designs, materials and techniques has been flourishing in recent decades, and such advances have led to an implant survival rate of approximately 95% according to 10-year clinical observations^2–5^. From this evidence, dental implants are considered an ideal option for functionally and aesthetically restoring missing tooth areas. However, conventional dental implants are somewhat out of step with patient-specific treatment strategies, necessitating additional surgical procedures, such as drilling or bone grafting.

Various efforts have been made to implement root analog implants to provide patient-specific dental treatment. The first attempt to apply a patient-specific root analog implant was made by Hodosh et al. in 1969^6^. They reported that collagenous fibers of the periodontal ligament inserted into the implant; however, when interpreted based on current histological knowledge, osseointegration failed and was considered to be fibrointegrated^7^. As the material was changed from polymethacrylate to titanium, root analog implant fabrication became possible, and numerous studies reported successful preclinical and clinical outcomes of root analog implants^7^.

With the advance of digital technologies and materials, elaborate 3D-printed personalized implant fabrication has become possible^8–10^. By virtue of the development of cone beam computed tomography (CT), oral scanning, and computer-aided design software, personalized 3D-printed implant structures can be manipulated and subsequently fabricated with additive manufacturing. A great number of studies have reported on 3D-printed implants displaying successful osseointegration and good biocompatibility in vivo^11–13^. In terms of the material, the sand-blasted 3D-printed Ti–6Al–4V specimen had similar biological properties in terms of adherent cell numbers, vinculin intensity, osteogenic gene expression and biomineralization to those of the machine-cut counterpart, indicating the potential usefulness of 3D printing technology in dental implant^14^. In vitro studies of 3D-printed Ti–6Al–4V implants also revealed that no harmful or adverse effects on cell proliferation or spreading, indicating that it is biocompatible. As expected, the surface micro/nano-structured implants outperformed the polished implants in terms of osteogenic differentiation at both the protein and gene levels^13^. In addition, Shaoki et al. demonstrated that 3D-printed implants had similar BV/TV values and BIC ratios to machined implants, even though cell adhesion, osteoblast differentiation, and removal torque were higher on the former^15^.

3D-printed implants with multiple roots in the posterior region have been suggested as an alternative to alleviate the mechanical complications of conventional implants and regenerative procedures. Recently, we investigated 3D-printed double-root implants designed under a digital workflow with digital data, software, and fabricated with a direct metal laser sintering machine using Ti–6Al–4V powder in vivo^16^. From this study, we found that the macrodesign of a 3D-printed implant with a groove has a significant positive effect on secondary implant stability.

However, a peculiarity was found in that the marginal bone changes in the furcation area were larger than those in the mesial or distal areas for lattice-type 3D-printed implants. As it is difficult to perform daily self-cleansing of furcation-involved teeth, bacterial accumulation progresses continuously, and the long-term prognosis of the teeth cannot be guaranteed^17^. Therefore, before implementing double-root implants in clinical situations, it is necessary to investigate the outcome of furcation-involved double-root implants and examine whether periodontal healthy conditions can be secured compared with single-root implants.

Therefore, the purpose of this study is to investigate the marginal bone changes of double-root 3D-printed implants using Ti–6Al–4V powder compared to single-root 3D-printed implants, in addition to implant stability and micro-CT and histological analyses.

## Materials and methods

**Figure 1:**
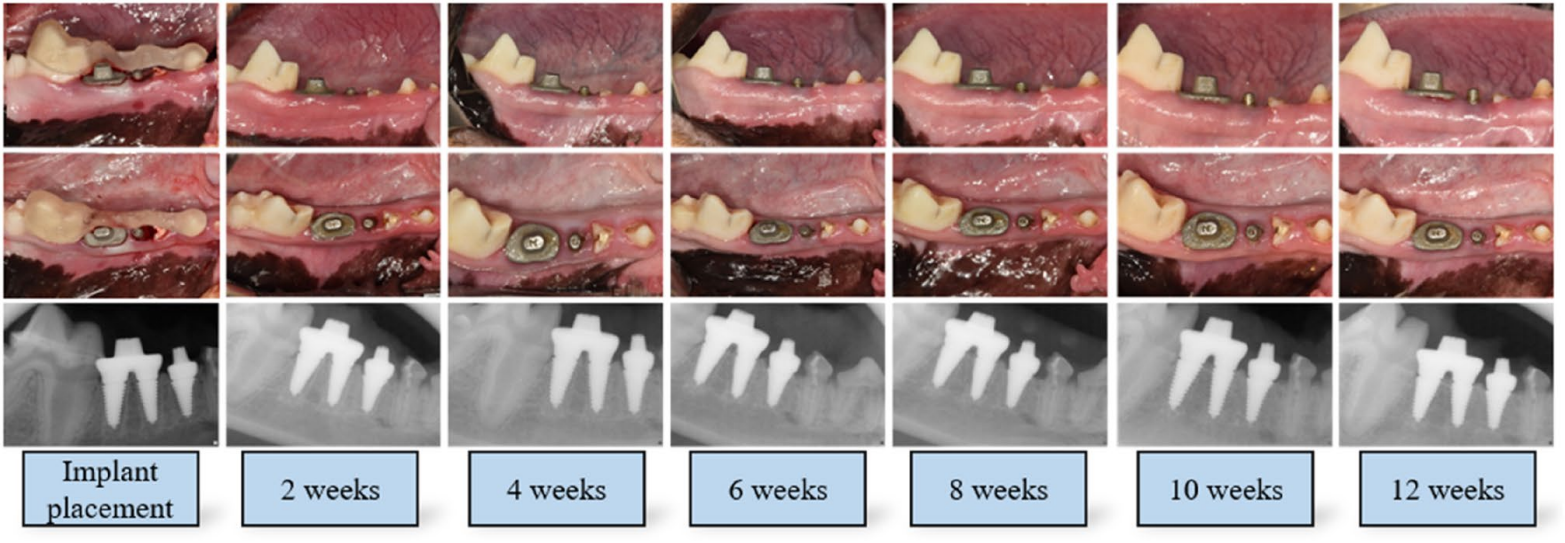
Clinical and radiographic photograph of single- and double-root 3D-printed implant. All protective caps were removed 2 weeks after implant placement for plaque control and implant stability measurements. There were no clinical signs of peri-implant inflammation, including redness, spontaneous bleeding, swelling, or ulceration. The clinical and radiographic photos were taken at the time of implant placement and 2, 4, 6, 8, 10, 12 weeks after implant placement.

All methods in this animal experiment were performed in conformity with the principles of the 3R (Replacement, Reduction, and Refinement) and two major laws in Korea which are Animal Protection Act established by the Ministry of Agriculture Food and Rural Affairs, and the Laboratory Animal Act established by the Ministry of Food and Drug Safety. The animal experiment was evaluated and authorized by the Institutional Animal Care and Use Committee of Seoul National University (IACUC; approval no. SNU-210115-–1) and performed in accordance with the Animal Research: Reporting of In Vivo Experiments (ARRIVE) guidelines. The study comprised four 1-year-old male beagle dogs, weighing approximately 10–12 kg. The manuscript was written in compliance with the ARRIVE guidelines. The timeline of this study is presented in Fig. 1.

### Fabrication of 3D-printed implants

The fabrication process of the 3D-printed implants was conducted according to a previous study^16^. In brief, CT datasets of the mandible were obtained using a CT scanner (GE, Boston, USA) and were imported into 3D reconstruction software (Materialise, Leuven, Belgium) via Digital Imaging and Communications in Medicine format. Both sides of the mandibular third and fourth premolars were virtually extracted and isolated as a stereolithography (STL) file with the software. The STL file was transferred into software (Materialise) to fabricate 3D single-root implants at the distal root of the third premolar area and double-root implants at the fourth premolar area (Fig. 1) with a direct metal laser sintering machine using Ti–6Al–4V powder through Dentium Build Processor 1.4.7 (Dentium, Seoul, Korea) powered by KETI Slicing Engine. The single-root implant and mesial root of the double-root implants were manufactured to be 2 mm longer than the corresponding teeth with a groove to obtain primary stability. The implants were marked with numbers and letters in the upper area to denote animals and locations. The root dimensions of the 3D-printed implants were different for each tooth, but the abutment was manufactured with a constant size. Following large-grit sandblasting and acid-etching (SLA) surface treatment according to a previous study, the 3D-printed implants were sterilized using gamma-ray irradiation, which emits short wavelength light from a cobalt-60 (60Co) radioactive isotope. A surgical guide and drills to perform osteotomies at the mesial roots of the planned sites were fabricated through digital light processing (DLP) 3D-printer (Dental 3DPrinter-P, Dentium, Seoul, Korea) using material Surgical Guide (DG-1). The protective cap was fabricated with polymer with a thickness of 1 mm to minimize loading on the implant (DG-1, Hephzibah, Inchon, Korea).

### Immediate placement of 3D-printed implants

The animals were anesthetized by a veterinarian using intravenous injections of tiletamine/zolazepam (5 mg/kg, Virbac, Carros, France), xylazine (2.3 mg/kg, Bayer Korea, Ansan, Korea), and 0.05 mg/kg atropine sulfate for the surgery. Complementary local anesthesia was injected at the mandibular third and fourth premolar area with 2% lidocaine HCl with epinephrine (1:1,000,000, Huons, Seongnam, Korea). The third and fourth premolars were hemisectioned with a diamond fissure bur in the buccolingual direction of the teeth and atraumatically extracted with elevator and forceps without flap reflection. The apical portion of the extraction socket was prepared using a 2.3 mm drill with a motor-driven handpiece (EXPERTsurg LUX, KaVo, Warthausen, Germany) to be 2 mm longer than the corresponding root for single-root 3D-printed implant, and mesial root for double-root 3D-printed implant. The 3D-printed implant heads were directly tapped using a surgical mallet. The protective cap was attached to the adjacent teeth using resin-modified glass ionomer cement (GC FujiCEM2, Tokyo, Japan).

### Postoperative care

An antibiotic (cefazoline, 20 mg/kg, Chongkundang Pharm., Seoul, Korea) and analgesic (tramadol hydrochloride, 5 mg/kg, Samsung Pharm., Hwaseong, Korea) were intravenously injected after surgery to relieve postoperative pain and inflammation. For 3 days after the surgery, antibiotics and analgesics were administered by mixing with the animals’ diet. To prevent any mechanical pressure that might hinder wound healing, a soft diet was provided for a month. The surgical sites were inspected every 2 weeks and rinsed with 0.12% chlorhexidine gluconate solution (Hexamedine, Bukwang Pharm., Seoul, Korea).

### Implant stability measurements

Based on a previous study^11,16,18^, damping capacity analysis (Anycheck, Neobiotech, Seoul, Korea) was performed at implant placement and at every two weeks following until 12 weeks to measure implant stability. Measurements were taken five times from the buccal side of each implant, and the average value was considered representative.

### Marginal bone changes

The marginal bone level was measured with periapical radiographs taken at implant placement, 6 weeks and 12 weeks followed by implant placement. The measurement was performed at the mesial and distal sites of each implant and middle sites in the case of double-root implants. Mesial and distal marginal bone loss at 6 weeks and 12 weeks were each compared between the two 3D-printed implant groups. The marginal bone loss of the double-root 3D-printed implants at 6 and 12 weeks was compared among mesial, middle, and distal sites.

### Micro-CT analyses

Animals were sacrificed 12 weeks after implant placement with potassium chloride (75 mg/kg, Jeil Pharm., Daegu, Korea). The block biopsy from each experimental site was harvested for micro-CT and histological preparation. The scan was performed at an energy of 60 kV, intensity of 167 μA, and resolution of 13.3 μm using a 0.5-mm aluminum filter and a 3-dimensional micro-CT machine (SkyScan 1172, SkyScan, Aartselaar, Belgium). The data were reconstructed with the manufacturer’s software (DataViewer 1.5.2.4 64-bit version, Bruker micro-CT, Skyscan, Kontich, Belgium) and quantitatively analyzed with CTAn (Bruker-CT, Kontich, Belgium). Based on a previous study^16^, the volume of interest (VOI) was set to a 190-μm circular band stretching 60–2250 μm from the implant surface of each root, limiting 1 mm to 4 mm above the fixture apex.

### Histological preparation

After 1 week in a fixative solution containing 10% neutral formalin buffer, the tissue sections were dehydrated in a series of ethanol solutions. Subsequently, the samples were embedded in methacrylate (Technovit 7200, Heraeus Kulzer, Hanau, Germany). The central mesiodistal sections were prepared and polished to approximately 45 ± 5 μm and stained with Goldner trichrome.

### Histological and histomorphometric analyses

Histological slides were stored as digital images after scanning with Panoramic 250 Flash III (3DHISTECH, Budapest, Hungary). The region of interest (ROI) was selected from 1 to 4 mm above the fixture apex using a computer-aided slide image analysis program (CaseViewer 2.2; 3DHISTECH Ltd., Budapest, Hungary). As described in a previous study^16^, bone-to-implant contact (BIC) and bone area fraction occupancy (BAFO) were measured from each 3D printed implant.

### Statistical analysis

A sample size calculation was not performed due to the pilot nature of the study. All data of the two types of 3D-printed implants are presented as the means ± SDs. Two-way ANOVA (implant type and time period) was conducted, and Sidak’s multiple comparisons test was performed for implant stability and marginal bone changes. An unpaired t test was conducted in the micro-CT analysis. Due to the lack of normality test passes, the Mann‒Whitney test was performed for BIC and BAFO.

## Results

### Clinical observations

All single- and double-root implants survived (Fig. 1). There were no clinical signs of peri-implant inflammation, including redness, spontaneous bleeding, swelling, or ulceration. All protective caps were removed 2 weeks after implant installation as described in a previous study^16^.

### Implant stability measurements

**Figure 2:**
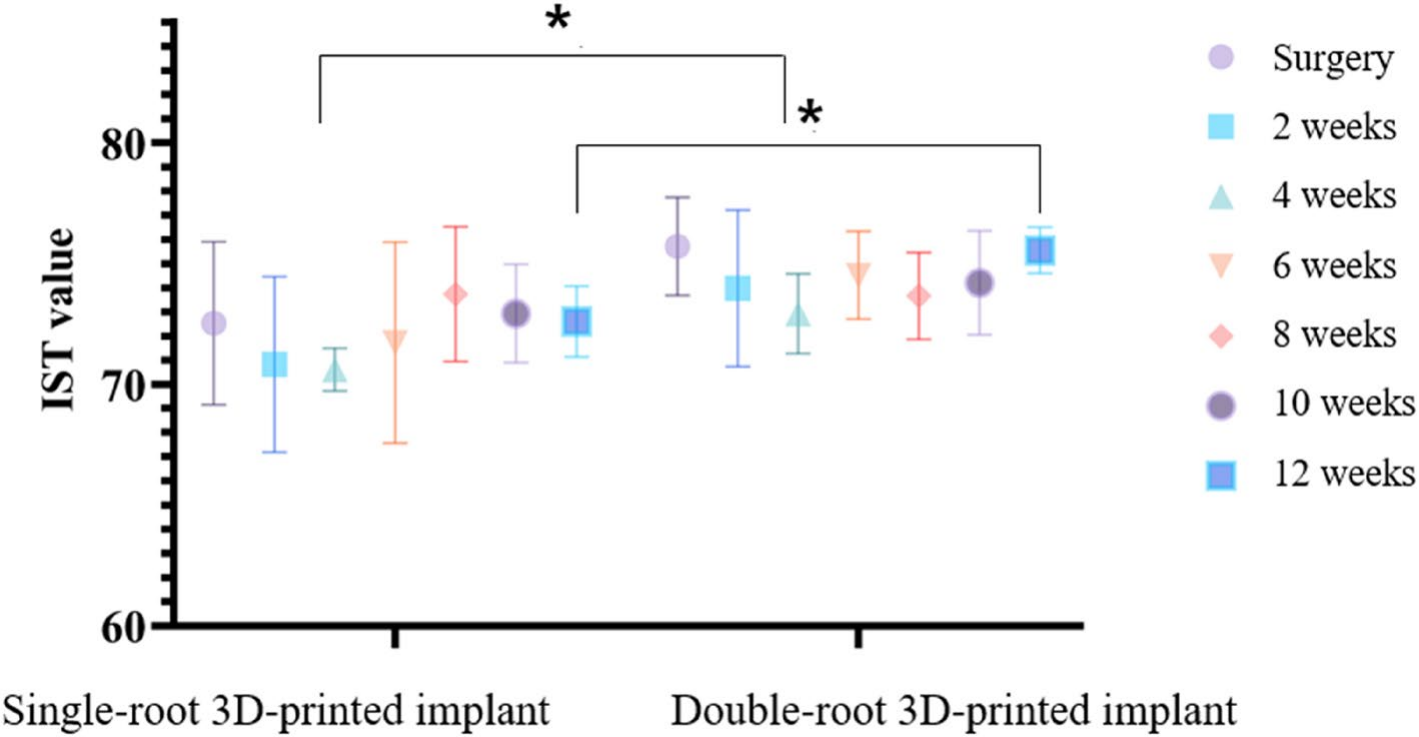
Implant stability test (IST) values of single- and double-root 3D-printed implants. The IST values were significantly greater for the double-root 3D-printed implants at 4 and 12 weeks than for the single-root 3D-printed implants, as shown by Sidak’s multiple comparison test (*p* = 0.0143, *p* = 0.0320). There were no significant differences within the group during each time point.

Implant stability values are presented in Fig. 2. The implant stability value of single-root 3D-printed implants was 72.53 ± 3.38 at the time of implant surgery and 70.83 ± 3.63, 70.60 ± 0.89, 71.73 ± 4.16, 73.73 ± 2.79, 72.93 ± 2.04, and 72.60 ± 1.46 at every 2 weeks after until 12 weeks following implant placement. The implant stability value of double-root 3D-printed implants was 75.71 ± 2.03 at implant placement and 73.97 ± 3.24, 72.92 ± 1.65, 74.51 ± 1.81, 73.65 ± 1.80, 74.20 ± 2.15, 75.54 ± 0.96 at 2 weeks after until 12 weeks following implant placement. There were no significant differences within the group during each time point, but statistically significant differences were observed between single- and double-root implants at 4 and 12 weeks (*p* = 0.0143 and 0.0320).

### Marginal bone changes

**Figure 3:**
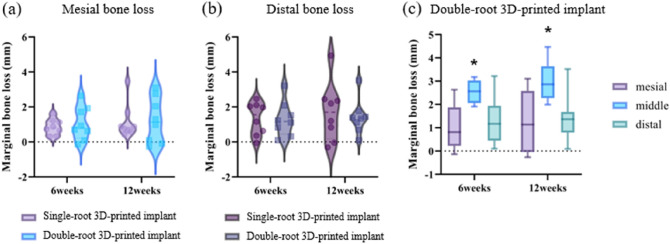
Radiographic changes (**a**) mesial bone loss in single- and double-root 3D-printed implant at 6 and 12 weeks (**b**) distal bone loss in single- and double-root 3D-printed implant at 6 and 12 weeks (**c**) Mesial, middle, and distal bone loss in double-root 3D-printed implant at 6 and 12 weeks. Asterisks (*) indicate statistically significant difference in marginal bone loss in the middle area compared with mesial and distal areas at 6 weeks and 12 weeks. 3D: 3-dimensional.

The marginal bone losses at the mesial sites of the single- and double-root 3D-printed implants were 0.85 ± 0.45 mm and 1.06 ± 0.95 mm, respectively, at 6 weeks. These values were 1.17 ± 1.00 mm and 1.24 ± 1.30 mm, respectively, at 12 weeks. No significant differences were observed in terms of the implant type or time point (Fig. 3a). The marginal bone losses at the distal sites of the single- and double-root 3D-printed implants were 1.33 ± 0.94 mm and 1.30 ± 0.99 mm at 6 weeks, respectively. The values were 1.70 ± 1.68 mm and 1.42 ± 0.99 mm at 12 weeks. No significant differences were observed in terms of implant type or time point (Fig. 3b).

The marginal bone losses at the mesial, middle, and distal sites of the double-root 3D-printed implants at 6 weeks were 1.06 ± 0.95 mm, 2.55 ± 0.50 mm, and 1.30 ± 0.99 mm, respectively. The marginal bone losses at the mesial, middle, and distal sites of the double-root 3D-printed implants at 12 weeks were 1.24 ± 1.30 mm, 3.00 ± 0.85 mm, and 1.42 ± 0.99 mm, respectively. The marginal bone loss at the middle site of the double-root 3D-printed implant at each time point showed higher values than that at the mesial and distal sites of the double-root 3D-printed implant (Fig. 3c).

### Micro-CT analyses

**Figure 4:**
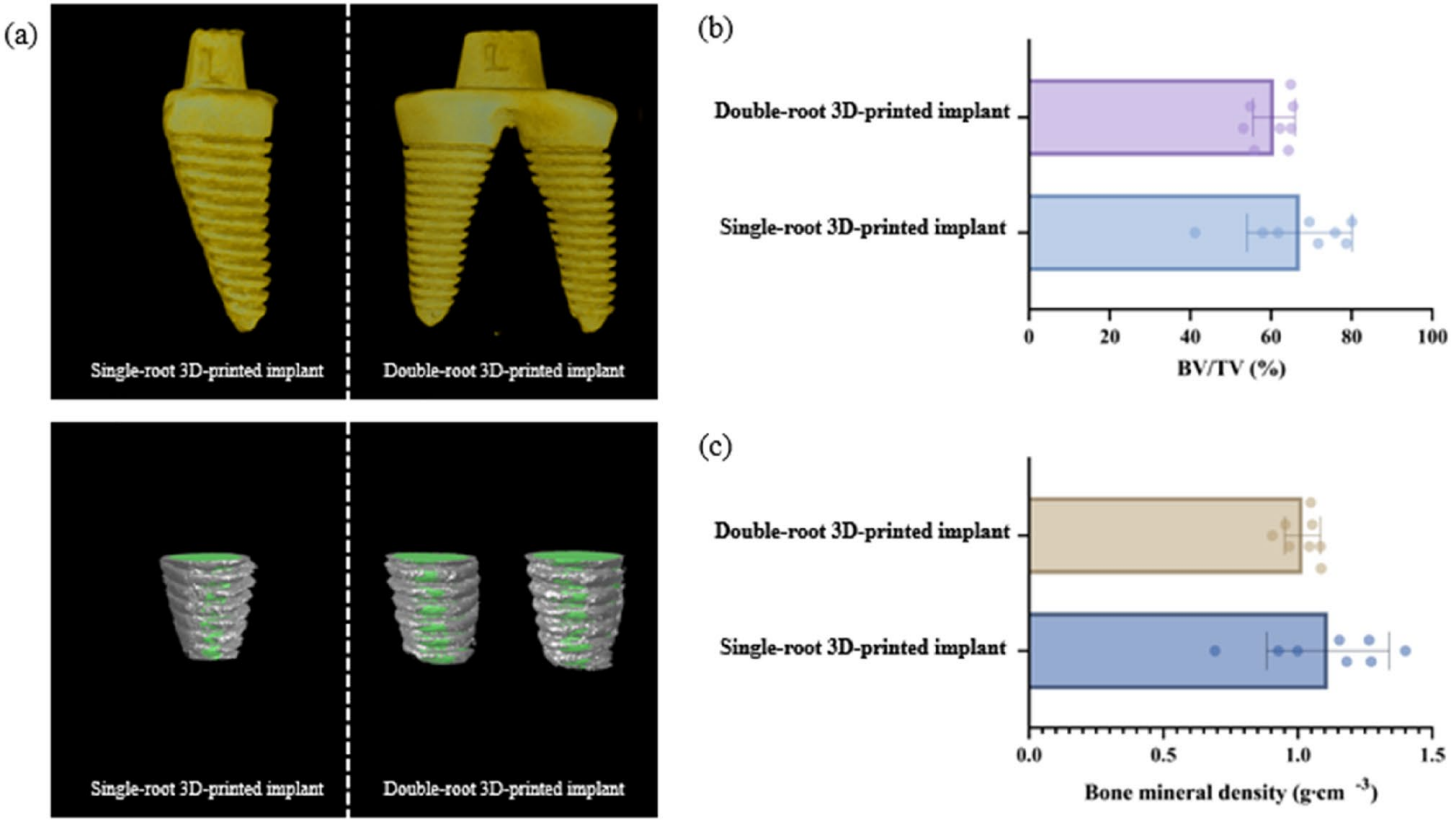
Representative micro-computed tomography (**a**) and analysis (**b**, **c**). (**a**) Gray and green areas indicate the VOI and 3D-printed implants, respectively. BMD and BV/TV were measured in the gray area. (**b**) The BV/TV of single-root and multi-root 3D printed implant were 67.11 ± 13.05% and 60.76 ± 5.43%, respectively, showing no statistically significant difference. (**c**) The bone mineral density of single-root and multi-root 3D printed implant were 1.11 ± 0.23 g mm^−3^ and 1.02 ± 0.08 g mm^−3^, respectively, showing no statistically significant difference. CT: computed tomography, VOI: volume of interest, 3D: 3-dimensional, BMD: bone mineral density, BV/TV: bone volume/tissue volume.

The results from the micro-CT analysis are described in Fig. 4. The BV/TV values of the single-root and double-root 3D-printed implants were 67.11 ± 13.05% and 60.76 ± 5.43%, respectively, showing no statistically significant difference. The bone mineral densities of the single-root and double-root 3D-printed implants were 1.11 ± 0.23 g mm^−3^ and 1.02 ± 0.08 g mm^−3^, respectively, showing no statistically significant difference.

### Histological observations

All eight single-root and eight double-root 3D-printed implants survived. There were no specific inflammation signs. Marginal bone loss at the mesial and distal sites was observed in both groups with no significant differences. A marginal bone loss pattern at the furcation area was observed for the double-root 3D-printed implants.

### Histomorphometric analyses

**Figure 5:**
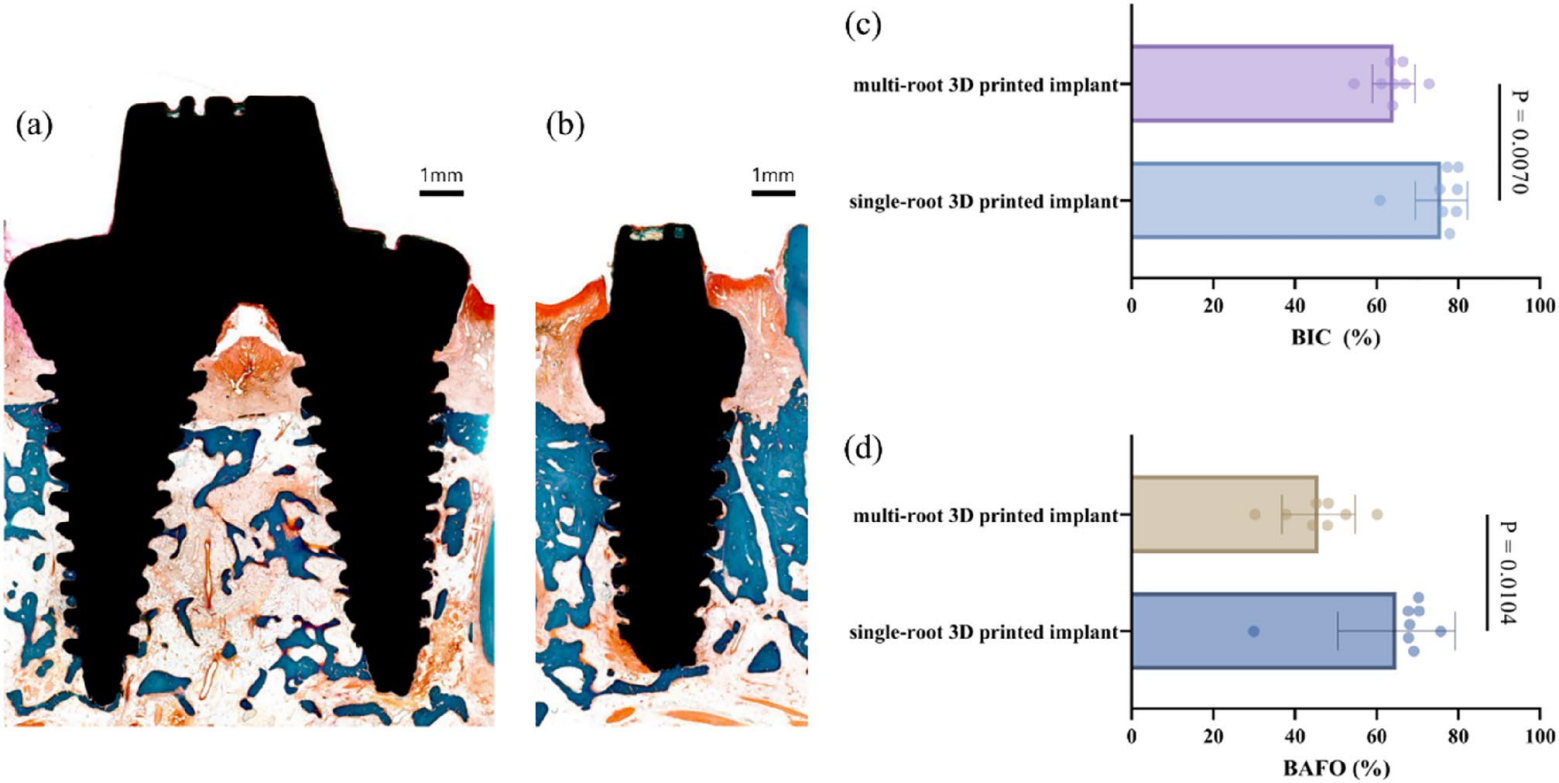
Representative of histologic view (**a**, **b**) and analysis (**b**, **d**). (**a**)Histomorphometric view of double-root 3D-printed implant. Note the bone resorption in the furcation area. (**b**) Histomorphometric view of single-root 3D-printed implant (**c**) The BIC in single-root and multi-root 3D-printed implants had statistically significant difference (75.87% ± 6.32% and 64.18 ± 5.23%, respectively, *p* = 0.0070) (**d**) The BAFO was statistically significantly different (*p* = 0.0104) in single-root and multi-root 3D-printed implants (64.88 ± 14.37% and 45.81 ± 9.01%, respectively). 3D: 3-dimensional, BIC: bone-to-implant contact, BAFO: bone area fraction occupancy.

The results from the micro-CT analysis are described in Fig. 5. The BIC values for the single-root and double-root 3D-printed implants were significantly different (75.87% ± 6.32% and 64.18 ± 5.23%, respectively, *p* = 0.0070). The BAFO values were significantly different (*p* = 0.0104) for the single-root and double-root 3D-printed implants (64.88 ± 14.37% and 45.81 ± 9.01%, respectively).

## Discussion

This study compared the implant stability, marginal bone loss, BV/TV, BMD, BIC, and BAFO values of single- and double-root 3D-printed implants using Ti–6Al–4V powder through micro-CT, histological and histomorphometric analyses. The double-root 3D-printed implants showed (i) greater implant stability, which was statistically significant at 4 and 12 weeks; (ii) comparable marginal bone loss at the proximal area but statistically greater marginal bone loss at the middle area; (iii) no significantly different BV/TV and BMD values through micro-CT analyses; and (iv) significantly lower BIC and BAFO values through histomorphometric analyses compared to single-root 3D-printed implants.

3D printing technology using Ti–6Al–4V have gained increased interest in implant dentistry in recent years. Despite its significant demand, manufacturing Ti6Al4V implant is difficult because of its low thermal conductivity^16^, tendency to strain harden^17,18^, and aggressive chemical reactivity to oxygen^19^. The conventional Ti–6Al–4V manufacturing technique relies on forging, casting, and rolling of bulk raw materials, followed by subsequent machining to final forms and dimensions; however, these processes invariably produce significant material waste, high manufacturing costs, and protracted lead times^20,21^. In such cases, additive manufacturing (AM), a modern 3D printing technique that creates near-net form structures directly from CAD models by adding materials layer-by-layer, offers its advantageous capability for production of Ti–6Al–4V products with geometric complexity. Therefore, due to its free design, single-piece customization, and great process efficiency meeting the urgent biomedical field needs, the clinical applications of 3D-printing piqued in recent years^13^.

Individualized designs may be created using the additive manufacturing method, and with macrodesign adjustments and surface treatments, the feasibility of using 3D-printed implants has been studied^12,19,20^. However, the evidence of pros and cons of double-root 3D-printed implants in comparison to the single-root 3D-printed implants is limited. This study found that double-root 3D-printed implants has comparable implant stability, but greater marginal bone loss due to the furcation area compared to the single-root 3D-printed implants.

The implant stability values at 4 and 12 weeks of the double-root implants showed higher values compared with those of the single-root implants. Most implant stability values were above 70, indicating that the implant was clinically stable for functional loading. The positive results of the two groups at all time points might stem from their groove structure, which resulted in high implant survival and stability as a macroretention structure for lattice-type 3D-printed implants in a previous study^16^. The values tended to be reduced at 2 and 4 weeks for each group, although that was not statistically significant within the groups. This tendency reflects the stability dip, which results in the lowest implant stability value during the early healing period, as reported in several studies^21–23^. As in previous studies, the difference in the implant stability value according to implant design appears to be most evident at the time of this stability dip^16,21–23^. The higher stability values at 4 weeks are probably due to the additional effect of the double-root structure on implant stability.

In radiographic assessments, the single- and double-root 3D-printed implants displayed similar marginal bone loss values at mesial and distal sites regardless of the implant type and time point. For the double-root 3D-printed implants, significantly higher marginal bone loss was observed at the furcation area (middle) at each time point. This result can be explained by previous studies showing that a narrow inter-implant distance (less than 3 mm) resulted in marginal bone loss^24^. A previous study demonstrated that an increase in the available space for inflammatory cell infiltrate can reduce marginal bone loss^25^. In our study, the inter-root distance was narrow in the upper area because the 3D-printed implants reflected the shape of the teeth as much as possible with their tapered morphology. Taken together, these findings show that a narrow inter-root space in the upper area seemed to result in marginal bone loss in the furcation area.

According to the accepted standards for assessing implant survival and success, the marginal bone level change in the first year should be less than 1.5 mm, and this concept is widely accepted^26^. The systematic review and meta-analysis by Ragucci et al.^27^ evaluated the marginal bone loss in the implants placed immediately in the extraction socket in the molar areas, including data of 372 implants in 11 studies. The marginal bone loss was estimated to be 1.29 ± 0.24 mm in over 1 year follow-up period with 95% CI (0.81–1.76). Although differences in the study design, study subject and implant material exist, the marginal bone loss found in the single-root 3D-printed implants of the current study (1.17 ± 1.00 mm in mesial and 1.24 ± 1.30 mm in distal area) is comparable to that of the previous studies. The marginal bone loss in the double-root 3D-printed implants show comparable marginal bone loss values at the mesial and distal sites at 12 weeks (1.24 ± 1.30 mm and 1.42 ± 0.99 mm, respectively) as well, but the value was statistically significantly greater at the middle site (3.00 ± 0.85 mm).

In micro-CT analyses, the BV/TV and BMD values of the single-root and double-root 3D-printed implants did not differ significantly. Therefore, it appears that the quantity and density of the bone around the 3D-printed implants are not affected by the number of roots of 3D-printed implants. The morphology of the implant fixture does not seem to affect peri-implant bone healing when an implant with the proper surface for osseointegration is kept stable. These results corroborate that Ti–6Al–4 V powder is a biocompatible material that is suitable for dental 3D-printed implants. This outcome is in line with our previous study in which implants fabricated with the same materials showed comparable BV/TV and BMD values^16^.

In histomorphometric analyses, the BIC and BAFO values were statistically lower for the double-root 3D-printed implants than for the single-root 3D-implants. This can probably be explained by the pattern in which the furcation area of the double-root implants exhibited significant interradicular bone resorption. Since BIC and BAFO refer to the amount or percentage of implant surface area in contact with the bone, the values are inevitably lower than those of single-root implants, which do not have the disadvantage of furcation. Although implant design with multiple roots in the posterior regions has been suggested to reduce the mechanical complications of conventional implants, the outcome of furcation-involved areas remains enigmatic.

In this study, prosthetic loading conditions with 3D-printed implants were analyzed only until the implant installation stage, not under prosthetic loading conditions. However, occlusion is one of the most significant factors affecting peri-implant hard tissue and implant success. In response to mechanical stress, occlusion can impact peri-implant hard tissue remodeling. From this perspective, future studies should incorporate comprehensive conditions to evaluate functional stability.

Another limitation of this study is the method of implant installation due to the shape of the double-root implants. The double-root 3D-printed implant has been incorporated to alleviate the mechanical complications of conventional implants and regenerative procedures, but the method of tapping the divergent double-root implant using a surgical mallet may increase strain within the bone. Although divergent roots distribute the occlusal force, the nature of the divergent root shape is a hindrance when placing the fixture into the bone. In addition, this process of placing the implant hinders the accurate positioning of the fixture apicocoronally and buccolingually, since the tapping vector and force cannot be precisely controlled. Further studies should investigate the effect of striking 3D implant fixtures into the alveolar bone and possible errors derived from the process.

Within the limitations of this preclinical study, 3D-printed double-root implants showed comparable stability, proximal marginal bone loss, BV/TV and BMD values compared with single-root implants. However, the double-root implants demonstrated significant marginal bone loss in the furcation area and lower BIC and BAFO values than single-root implants.

## Data Availability

The datasets used and/or analyzed during the current study are available from the corresponding authors on reasonable request.
